# Canadian undergraduate men’s visual attention to cisgender women, cisgender men, and feminine trans individuals

**DOI:** 10.1038/s41598-020-79870-2

**Published:** 2021-01-11

**Authors:** Lanna J. Petterson, Paul L. Vasey

**Affiliations:** Laboratory of Comparative Sexuality, Department of Psychology, University of Lethbridge, 4401 University Drive, Lethbridge, AB T1K 3M4 Canada

**Keywords:** Psychology, Human behaviour

## Abstract

Some heterosexual men express sexual interest in feminine trans individuals with penises. It is possible that this interest arises from a tendency for heterosexual men to be sexually responsive to gender in addition to sex. We compared the self-reported sexual attraction and visual attention patterns of Canadian undergraduate heterosexual men (*N* = 51) and gay men (*N* = 20) to nude images of feminine trans individuals with penises, cisgender men, and cisgender women. Heterosexual men were most attracted to cisgender women and fixated on them the longest. However, they were more attracted to feminine trans individuals with penises than to cisgender men. They also biased their attention to feminine trans individuals with penises over cisgender men. This pattern was unique to heterosexual men. Gay men were most attracted to cisgender men and allocated the most visual attention to them. They responded to feminine trans individuals and cisgender women in a relatively similar manner. As such, heterosexual men appear to be responsive to sex and gender, which may account for sexual interest in feminine trans individuals among some heterosexual men.

Transgender refers to individuals whose gender (i.e., presentation and identity) does not conform to the one they were assigned at birth. The term *feminine trans individuals* will be used to refer to those who were assigned a male-typical sex and gender at birth but who present in a feminine manner, either continuously or periodically. The identities of feminine trans individuals vary both within and between cultures^1–3^. Whereas some of the individuals in question identify as *women* or *trans women*, particularly in Western and other industrialized societies, others may, for example, identify as a non-binary gender (i.e., one that is neither man nor women) or as gender fluid.

Heterosexual men’s willingness to engage in sexual interactions with feminine trans individuals who have penises appears to be highly variable across cultures^4^. Ethnographic research suggests that, in many cultures, these interactions are not uncommon^1,3^. In certain cultures, such as the USA and Canada, however, they appear to be relatively rare^5,6^. Across cultures, gay men are less likely than heterosexual men to express sexual interest in feminine trans individuals, including those with penises^7–10^.

The frequency with which feminine trans individuals augment their bodies using hormones and surgical procedures is also culturally varied. For example, in many cultures, most feminine trans individuals behave and dress in a traditionally female-typical manner but do not augment their bodies substantially beyond modifications such as shaving, growing out their hair, and wearing makeup^3^. In other cultures, such as Western ones, it is not uncommon for these individuals to use surgical and hormonal procedures for breast augmentation, facial feminization, and other body modification^11^.

Despite the substantial cross-cultural variability that characterizes men’s sexual behavior with feminine trans individuals, it is possible that the psychological predisposition to experience sexual interest in these individuals is cross-culturally universal^4^. Namely, heterosexual men may generally have the capacity to become sexually interested in feminine trans individuals because they share physical and behavioral characteristics with cisgender women. If so, predominantly heterosexual men would be predicted to exhibit sexual interest in feminine trans individuals that, although lower than their sexual interest in cisgender women, exceeds their sexual interest in cisgender men and non-sexual stimuli, regardless of cultural setting. In addition, heterosexual men would be predicted to exhibit significantly greater sexual interest in feminine trans individuals with breasts than those without breasts, the former having more physical characteristics in common with their preferred sexual targets than the later. Lastly, because heterosexual men’s interest in feminine trans individuals is hypothesized to arise from their sexual interest in cisgender women, this pattern should be unique to heterosexual men and not gay men (i.e., those who are sexually attracted to adult men).

To investigate these possibilities, we examined Canadian undergraduate heterosexual and gay men’s self-reported sexual attraction and visual attention to trans individuals who were feminine (e.g., wore make-up, female-typical hairstyles, and were posed in a feminine manner) and who had surgically augmented their breasts in a female-typical manner (hereafter, *feminine trans individuals with breasts*) as well as trans individuals who were feminine but who did not have augmented breasts (hereafter, *feminine trans individuals without breasts*). Feminine trans individuals with breasts appeared to have undergone other feminizing hormonal and surgical treatments (e.g., many had female-typical fat distributions and female-typical jaw lines, features which few feminine trans individuals possess without hormones and surgery), although none had undergone genital surgery. Responses to cisgender women, cisgender men, and non-sexual controls (bonobos, *Pan paniscus*, a species of great apes) were used for comparison. All of the humans depicted in these images were nude. To assess biases in visual attention, we employed a forced attention paradigm in which individual images were presented on opposite sides of the screen. Doing so precluded participants from viewing both images simultaneously. Previous research demonstrates that men bias their attention toward stimuli of their preferred gender and away from their non-preferred gender when using this paradigm^12–14^. In addition, we assessed whether sociosexuality (i.e., a preference for shorter-term and/or lower commitment relationships versus longer-term and/or higher commitment relations), interest in visual sexual stimuli, and homonegativity (i.e., negative attitudes toward same-sex attracted people) had a confounding influence on men’s self-reported visual attention patterns.

Specifically, we predicted that heterosexual men would report greater sexual attraction and allocate greater visual attention to cisgender women than to individuals with penises and other aspects of male-typical morphology (i.e., cisgender men and feminine trans individuals). However, heterosexual men were predicted to report greater sexual attraction and allocate greater visual attention to feminine trans individuals than to cisgender men and non-sexual stimuli. Furthermore, heterosexual men were predicted to report greater sexual attraction and allocate greater visual attention to feminine trans individuals with breasts than to feminine trans individuals without breasts. Finally, we predicted that, if the observed patterns reflect men’s sexual interest in women, they should be unique to heterosexual men.

## Methods

### Participants

Heterosexual and gay identified men were recruited from a small Canadian University. Psychology undergraduates were recruited through a participant research pool system (Sona). Participants were given 1% course credit. Gay men were recruited from the university through Sona for an additional semester and posters advertisements. Students recruited through posters were given $10 CAD.

Participants were required to have normal/corrected vision, identify as a heterosexual or gay man, and be over the age of 18. Men who did not identify as heterosexual or gay (e.g., bisexual men, pansexual men) were not included in the present study because they differ from heterosexual and gay men in their responses to images of cisgender women and men^15^. Additionally, participants were required to have viewed pornography and be comfortable viewing sexual imagery. The latter criteria were included to ensure that participants would be comfortable during the task and to minimize the likelihood of participants responding in a unique fashion due to discomfort or first exposure to sexual imagery. Ten participants were excluded from the visual attention analyses due to low gaze accuracy (< 80%), five were excluded from self-reported attraction analyses because they declined to answer or gave low ratings to all images, and two were excluded from description and analysis of questionnaire measures because they had duplicate participant numbers.

In total, 52 heterosexual undergraduate men (*M*_age_ = 22.2, *SD*_age_ = 3.94; age range = 18–32) and 20 gay undergraduate men (*M*_age_ = 22.7, *SD*_age_ = 5.50; age range = 18–37) were included in at least one analysis (all reported that their biological sex was male). Participants were grouped based on their sexual orientation identity. Analysis of self-reported sexual attraction pertained to 51 heterosexual men and 19 gay men. Analysis of visual attention pertained to 47 heterosexual men and 18 gay men. For the main analyses of visual attention, we had 80% power to detect a *R*^2^ value of 0.20 or greater among heterosexual men and a *R*^2^ value of 0.45 or greater among gay men^16^.

### Stimuli

Participants were shown 40 paired images, which included (1) nude feminine trans individuals with breasts, (2) nude feminine trans individuals without breasts, (3) nude women who appeared to be cisgender (hereafter, cisgender women), (4) nude men who appeared to be cisgender (hereafter cisgender men), and (5) bonobos (16 of each). Each stimulus category was matched with the remaining four categories. A fixation cross was located in the center of the screen and the images appeared in opposing corners (top right/bottom left or top left/bottom right). The images within each category pair appeared in a different corner of the screen each time that the pair was shown.

Nude images were taken from freely accessible websites using similar search terms (e.g. “hot” and “sexy”). Backgrounds were removed leaving only the model and images were transformed to grey scale^17^. Light intensity was adjusted to limit its effect on gaze patterns^18^. Additionally, mean bytes persevered after JPEG compression were compared^19,20^ because image complexity can influence ratings of image valence^21^. Individual images were adjusted to 450 pixels (or 6.25 inches) high with a resolution of 72 pixels/inch. Images ranged in width from 192 to 615 pixels. Some features could not be controlled between the human and bonobos: bonobos are darker than humans and their fur adds complexity. As such, the target images were similar in terms of their low-level features, but the neutral images contained some unique low-level features.

### Apparatus

The study was conducted using a 17-inch laptop with 1920 × 1080 resolution, a Tobii X3-120 Eye Tracker, and Tobii Pro Studio Software. The Tobii X3-120 uses near-infrared light operating at 120 Hz to illuminate the eyes and sensors capture pupil movement using bright and dark pupil detection. The X3-120 records participants’ distance from the screen and allows for small movements. The eye-tracker was calibrated for each participant, which required participants focus on 9 points on the screen.

### Procedure

Participants were told that the study’s purpose was to understand how people pay attention when evaluating the sexual appeal of humans who vary in terms of their gender presentation. This prompt was intentionally vague to avoid drawing participants’ attention to particular body regions of the images. Participants were informed that they would be required to evaluate the sexual appeal of nude images of men, women, and transgender individuals as well as bonobos.

During the study, participants were shown the series of paired images. Each pair remained on the screen for 10 s and was preceded by a fixation cross that appeared on the screen for 2 s. The image pairs were entered in a random order, but all participants saw the same order. After each image pair, participants were asked to identify and rate the sexual attractiveness of the image on the top and the image on the bottom. Following the experiment portion, participants were given a questionnaire.

### Measures and data treatment

#### Self-reported sexual attraction and image identification

Participants were asked to identify each image (response options: woman, man, transgender woman, or ape) and then rate how sexually attracted they were to each image using a 7-point Likert-type scale (response range: 1 = “not at all sexually attracted” to 7 = “extremely sexually attracted”). Image ratings were standardized within each participant. Mean values were calculated for each image category.

#### Attentional measures

Visual attention was evaluated using a forced attention paradigm^12–14,22^. Fixation patterns were defined using Tobii Pro Studio Fixation Filter. Initial attention was assessed using time to first fixation (TFF) on an image (low TFF scores indicate quicker attention capture). Controlled attention was assessed using total fixation duration (TFD) and total fixation count (TFC) for each stimuli category (high TFD and TFC scores indicate greater controlled attention). Values were winsorized to reduce the influence of outliers (values corresponding to a z-score > 3.29). Image values were standardized within each participant for all attention measures. Mean values were calculated for each image category.

#### Sexual orientation

Participants were asked whether they identified as heterosexual, gay, bisexual, or other (in which case, they were asked to specify their identity). In addition, participants’ reported their sexual behavior and sexual feelings (i.e., sexual attraction, thoughts, or fantasies) for men, women, and transgender women over their lifetime and the past year (response range: 0 = “no sexual activity with [sexual feelings for] [either women, men, or transgender women]” to 3 = “sexual activity with [feelings for] [either women, men, or transgender women] only”; X = “no sexual behaviour [sexual attractions, thoughts or fantasies]”).

#### Additional scale measures

Participants completed the Interest in Visual Sexual Stimuli Scale^23^, the Sociosexual Orientation Inventory^24^, and the Modern Homonegativity Scale^25^. Responses to the Interest in Visual Sexual Scale and the Modern Homonegativity Scale were averaged across each scale. Aggregate Sociosexual Orientation Inventory scores were calculated using the weighting suggested by Simpson and Gangestad^24^ (the sexual behavior items were capped at 30). Response to the scale measures were standardized across participants.

### Statistical analysis

Statistical analysis was conducted using RStudio, version 1.1.383^26^. The threshold for statistical significance was set at *p* < 0.005 whereas *p*-values below *p* < 0.05 were taken as suggestive evidence^27^. Due to the low power to detect interaction effects, sexual orientation groups were assessed separately. Gay men’s responses were used to assess whether the pattern found among heterosexual men was uniquely associated with male heterosexuality.

Non-parametric tests were used for analyses of sexual attraction ratings because participants’ responses were skewed. Three paired Wilcoxon tests with continuity correction were used to compare sexual attraction to (1) cisgender women and the grand mean of individuals with penises (i.e., feminine trans individuals and cisgender men), (2) cisgender men and the grand mean of feminine trans individuals, and (3) feminine trans individuals without breast and feminine trans individuals with breasts.

Analyses of visual attention were conducted using linear regressions. Three orthogonal contrasts were used to compare (1) cisgender women (coded as − 3) and individuals with penises (each coded as 1), (2) cisgender men (coded as − 2) and feminine trans individuals (each coded as 1), and (3) feminine trans individuals with breasts (coded as 1) and feminine trans individuals without breasts (coded as − 1) (categories that were not included in a contrast were coded as 0). Additionally, four planned contrasts were used to compare responses to bonobo and human images (for each contrast, the relevant human category was coded as 1 and all others were coded as 0; bonobos were always coded as 0).

Linear regressions with interest in visual sexual stimuli, sociosexuality, and homonegativity predicting visual attention were conducted. Orthogonal contrasts were created to compare (1) cisgender women and individuals with penises, (2) cisgender men and feminine trans individuals, and (3) response to feminine trans individuals without breasts and feminine trans individuals with breasts. For each measure, difference scores were included as separate dependent variables.

### Ethics statement

This research was approved by the University of Lethbridge Human Subjects Research Ethics Committee (#2016-108). All procedures performed were in accordance with the ethical standards of the University of Lethbridge and the Canadian Tri-Council Policy Statement: Ethical Conduct for Research Involving Humans (2018). Participants were required to provide written informed consent prior to participating. Participants were also required to provide verbal consent to allow their data to be used after completing the study.

## Results

**Figure 1:**
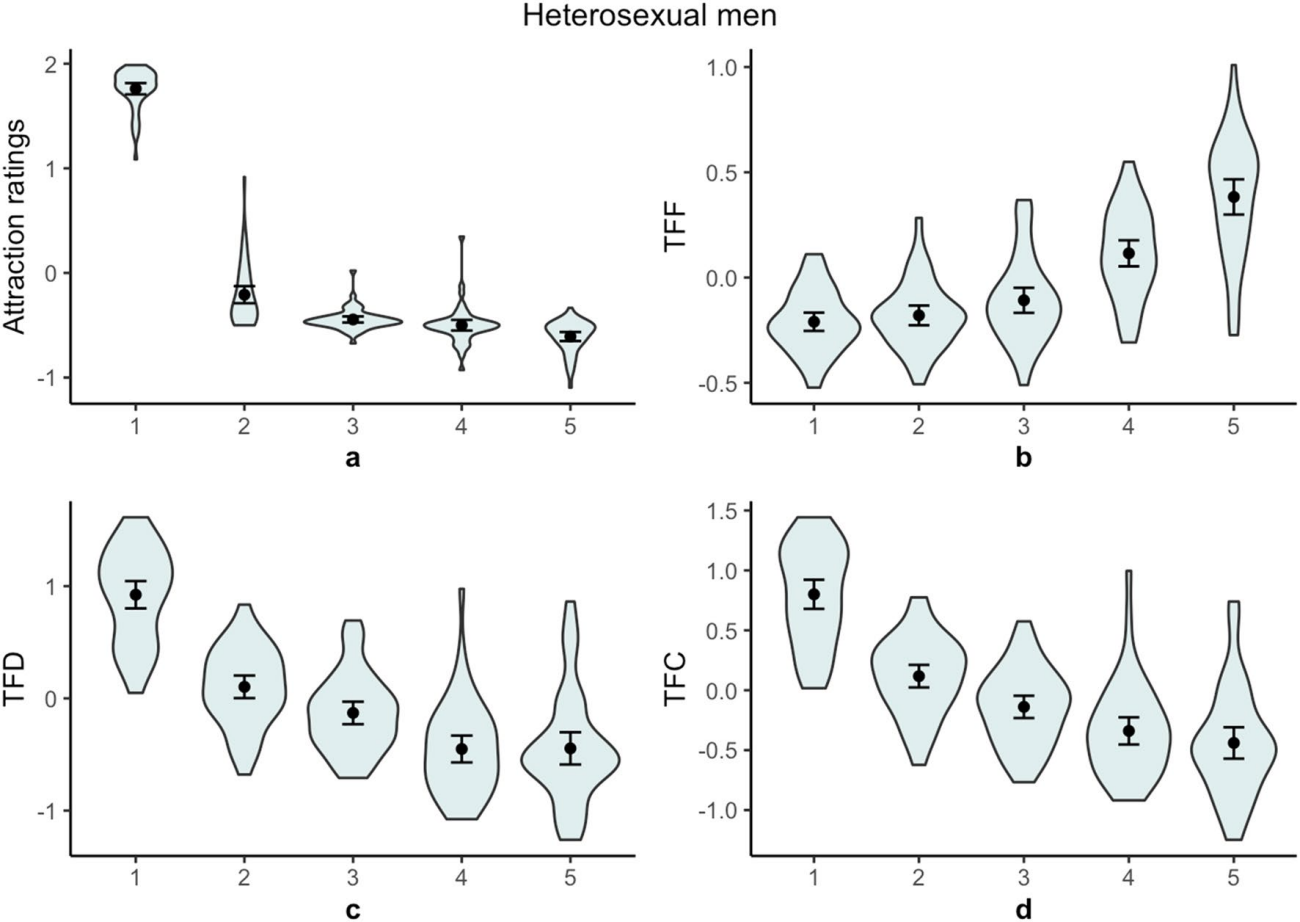
Heterosexual men’s (**a**) standardized attraction ratings, (**b**) standardized time to first fixation (TFF), (**c**) standardized total fixation duration (TFD), and (**d**) standardized total fixation count (TFC) by stimuli category. 1 = cisgender women, 2 = feminine trans individuals with breasts, 3 = feminine trans individuals without breasts, 4 = cisgender men, 5 = bonobos. Points indicate mean values. Capped lines show 95% confidence intervals. Shapes show the density of data points.

**Figure 2:**
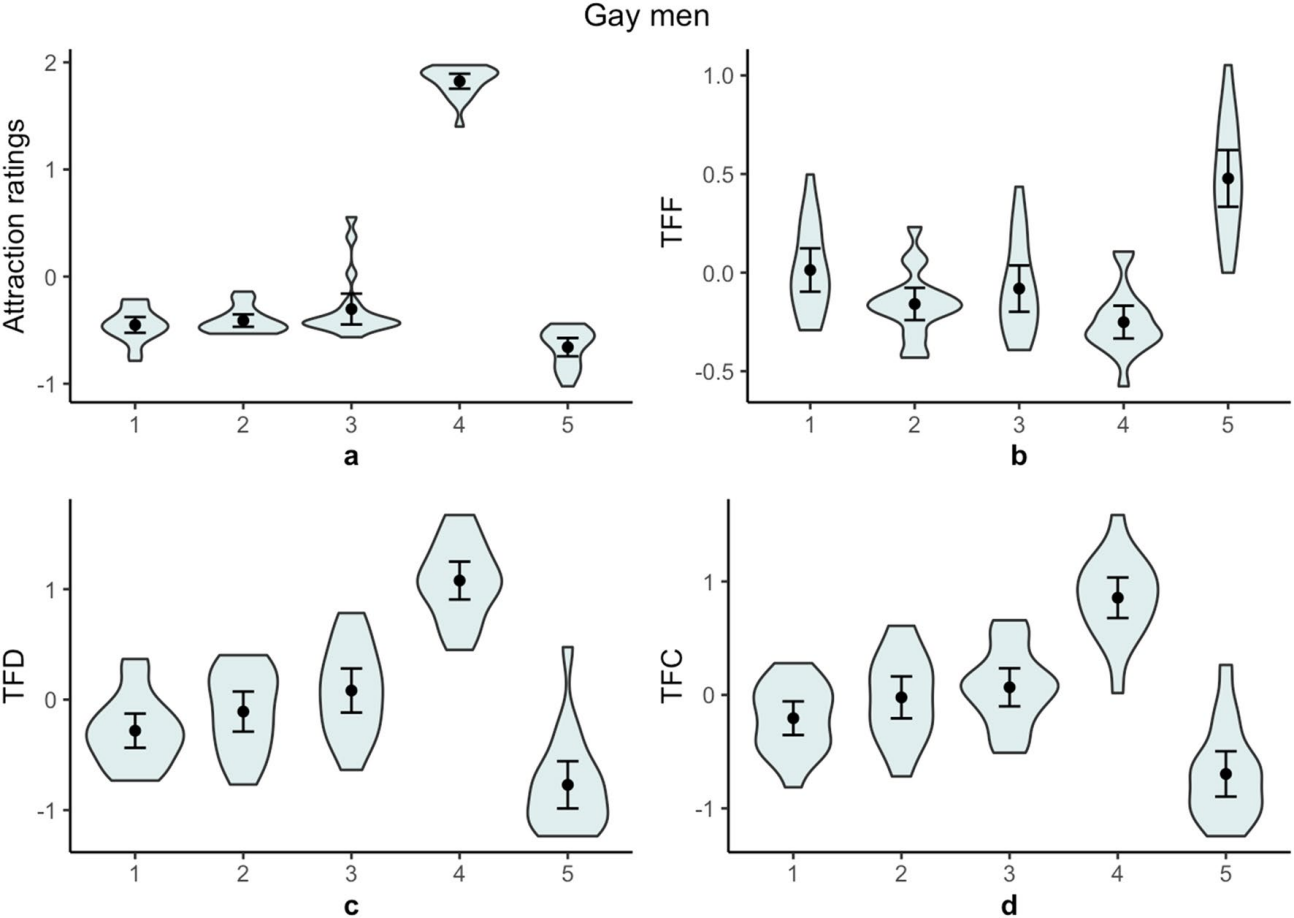
Gay men’s (**a**) standardized attraction ratings, (**b**) standardized time to first fixation (TFF), (**c**) standardized total fixation duration (TFD), and (**d**) standardized total fixation count (TFC) by stimuli category. 1 = cisgender women, 2 = feminine trans individuals with breasts, 3 = feminine trans individuals without breasts, 4 = cisgender men, 5 = bonobos. Points indicate group mean. Capped lines show 95% confidence intervals. Shapes show the density of data points.

Self-reported sexual attraction and behavior with transgender women, cisgender women, and cisgender men are shown in Table 1. Descriptive statistics for the standardized and raw self-reported sexual attraction, time to first fixation (TFF), total fixation duration (TFD), and total fixation count (TFC) measures are shown in Table 2. Figure 1 shows heterosexual men’s standardized attraction ratings and fixation patterns. Figure 2 shows gay men’s standardized attraction ratings and fixation patterns.

**Table 1:** Self-reported sexual behavior and feelings.

	Women	Men	Women and men	Women and transgender women	Men and transgender women	Women, men, and transgender women	No one
**Past year sexual behaviour: % (n)**							
Heterosexual men^a^	86 (43)	0	0	0	0	0	14 (7)
Gay men^b^	0	90 (18)	5 (1)	0	0	0	5 (1)
**Lifetime sexual behaviour: % (n)**							
Heterosexual men^a^	92 (46)	0	2 (1)	0	0	0	6 (3)
Gay men^b^	0	60 (12)	35 (7)	0	0	5 (1)	0
**Past year sexual feelings: % (n)**							
Heterosexual men^a^	78 (39)	0	6 (3)	12 (6)	0	4 (2)	0
Gay men^b^	0	50 (10)	25 (5)	0	20 (4)	5 (1)	0
**Lifetime sexual feelings: % (n)**							
Heterosexual men^a^	70 (35)	0	6 (3)	18 (9)	0	6 (3)	0
Gay men^b^	0	40 (8)	35 (7)	0	10 (2)	15 (3)	0

^a^*N* = 50.

^b^*N* = 20.

**Table 2:** Descriptive statistics for self-reported sexual attraction, time to first fixation, total fixation duration, and total fixation count.

	Cisgender Women	Feminine trans individuals with breasts	Feminine trans individuals without breasts	Cisgender men	Bonobos
**Standardized values **^**a**^					
Sexual attraction ratings: Mdn (SD)					
Heterosexual men: *N* = 51	1.80 (.19)	− .31 (.29)	− .46 (.10)	− .50 (.18)	− .57 (.15)
Gay men: *N* = 19	− .45 (.15)	− .43 (.12)	− .42 (.30)	1.86 (.14)	− .59 (.18)
Time to first fixation: M (SD)					
Heterosexual men: *N* = 47	− .21 (.15)	− .18 (.16)	− .11 (.20)	.12 (.21)	.38 (.29)
Gay men: *N* = 18	.01 (.22)	− .16 (.16)	− .08 (.24)	− .25 (.17)	.48 (.29)
Total fixation duration: M (SD)					
Heterosexual men: *N* = 47	.92 (.41)	.10 (.34)	− .13 (.34)	− .45 (.41)	− .45 (.49)
Gay men: *N* = 18	− .28 (.31)	− .11 (.36)	.08 (.40)	1.08 (.34)	− .77 (.43)
Total fixation count: M (SD)					
Heterosexual men: *N* = 47	.80 (.41)	.12 (.32)	− .14 (.32)	− .34 (.39)	− .44 (.45)
Gay men: *N* = 18	− .21 (.30)	− .02 (.37)	.07 (.34)	.86 (.36)	− .70 (.40)
**Raw values**^**a**^					
Sexual attraction ratings: Mdn (SD)					
Heterosexual men: *N* = 51	5.13 (1.03)	1.38 (.80)	1.13 (.41)	1 (.37)	1 (.04)
Gay men: *N* = 19	1.25 (.44)	1.25 (.53)	1.31 (.76)	6 (.88)	1 (.07)
Time to first fixation: M (SD)					
Heterosexual men: *N* = 47	1.04 (.49)	1.05 (.43)	1.14 (.58)	1.34 (.59)	1.62 (.66)
Gay men: *N* = 18	1.09 (.39)	.90 (.24)	.97 (.35)	.80 (.21)	1.51 (.42)
Total fixation duration: M (SD)					
Heterosexual men: *N* = 47	5.94 (1.45)	4.15 (1.22)	3.63 (1.18)	2.98 (1.14)	3.07 (1.07)
Gay men: *N* = 18	3.46 (1.04)	3.82 (1.14)	4.32 (1.01)	6.66 (1.10)	2.44 (.95)
Total fixation count: M (SD)					
Heterosexual men: *N* = 47	18.01 (6.22)	13.37 (4.97)	11.72 (4.98)	10.08 (3.87)	9.88 (3.77)
Gay men: *N* = 18	11.59 (4.25)	12.56 (4.31)	13.77 (6.21)	19.08 (7.45)	9.01 (5.05)

^a^Outlying time to first fixation, total fixation duration, and total fixation count values have been winsorized.

### Self-reported sexual attraction

Heterosexual men were more sexually attracted to cisgender women than to individuals with penises, *Z* = 6.21, *p* < 0.001, *r* = 0.87. They were less sexually attracted to cisgender men than to feminine trans individuals, *Z* = 4.58, *p* < 0.001, *r* = 0.64. Additionally, they were less sexually attracted to feminine trans individuals without breast than to feminine trans individuals with breasts, *Z* = 5.11, *p* < 0.001, *r* = 0.72.

Gay men were less sexually attracted to cisgender women than to individuals with penises, *Z* = 3.80, *p* < 0.001, *r* = 0.87. They were more sexually attracted to cisgender men than to feminine trans individuals, *Z* = 3.80, *p* < 0.001, *r* = 0.87. Gay men reported similar sexual attraction to feminine trans individuals without breast and feminine trans individuals with breasts, *Z* = 1.53, *p* = 0.127, *r* = 0.35.

### Time to first fixation

For heterosexual men, there was an effect of human-stimuli category on TFF, *F*(3, 184) = 30.58, *p* < 0.001, *R*^2^ = 0.33. They were slower to fixate on individuals with penises than on cisgender women, *b* = 0.04, 95% CI (0.02, 0.05), *SE* = 0.01, *p* < 0.001. However, they were quicker to fixate on feminine trans individuals than on cisgender men, *b* = − 0.09, 95% CI (− 0.11, − 0.06), *SE* = 0.01, *p* < 0.001. Their TFFs were similar for feminine trans individuals with breasts and feminine trans individuals without breasts, *b* = − 0.04, 95% CI (− 0.07, < 0.01), *SE* = 0.02, *p* = 0.058. The confidence intervals for heterosexual men’s TFFs on cisgender women, feminine trans individuals with breasts, and feminine trans individuals without breasts overlapped considerably, indicating that all feminine images tended to capture their early attention.

For gay men, there was an effect of human-stimuli category on TFF, *F*(3, 68) = 5.70, *p* = 0.002, *R*^2^ = 0.20. Gay men were quicker to fixate on individuals with penises than on cisgender women, *b* = − 0.04, 95% CI (− 0.07, − 0.02), *SE* = 0.01, *p* = 0.002. There was suggestive evidence that gay men were slower to fixate on feminine trans individuals than on cisgender men, *b* = 0.04, 95% CI (< 0.01, 0.08), *SE* = 0.02, *p* = 0.026. Gay men’s TFFs were similar for feminine trans individuals with breasts and feminine trans individuals without breasts, *b* = − 0.04, 95% CI (− 0.11, 0.03), *SE* = 0.03, *p* = 0.247.

There was an effect of stimuli category (including control images) on TFF for heterosexual men, *F*(4, 230) = 68.06, *p* < 0.001, *R*^2^ = 0.54, and gay men, *F*(4, 85) = 29.88, *p* < 0.001, *R*^2^ = 0.58. Heterosexual men and gay men were slower to fixate on images of bonobos than all other stimuli (all *p* values < 0.001).

### Total fixation duration

For heterosexual men, there was an effect of human-stimuli category on TFD, *F*(3, 184) = 113.50, *p* < 0.001, *R*^2^ = 0.65. Heterosexual men fixated on individuals with penises for less time than they fixated on cisgender women, *b* = − 0.27, 95% CI (− 0.30, − 0.24), *SE* = 0.02, *p* < 0.001. They fixated on feminine trans individuals longer than they fixated on cisgender men, *b* = 0.15, 95% CI (0.10, 0.19), *SE* = 0.02, *p* < 0.001. Additionally, they fixated on feminine trans individuals with breasts longer than they fixated on feminine trans individuals without breasts, *b* = 0.12, 95% CI (0.04, 0.19), *SE* = 0.04, *p* = 0.003.

For gay men, there was an effect of human-stimuli category on TFD, *F*(3, 68) = 52.34, *p* < 0.001, *R*^2^ = 0.70. Gay men fixated on individuals with penises longer than they fixated on cisgender women, *b* = 0.16, 95% CI (0.11, 0.21), *SE* = 0.02, *p* < 0.001. They fixated on feminine trans individuals for less time than they fixated on cisgender men, *b* = − 0.36, 95% CI (− 0.43, − 0.30), *SE* = 0.03, *p* < 0.001. Additionally, they fixated on feminine trans individuals with breasts and feminine trans individuals without breasts for a similar length of time, *b* = − 0.10, 95% CI (− 0.21, 0.02), *SE* = 0.06, *p* = 0.114.

There was an effect of stimuli category (including control images) on TFD for heterosexual men, *F*(4, 230) = 92.92, *p* < 0.001, *R*^2^ = 0.62, and gay men, *F*(4, 85) = 60.08, *p* < 0.001, *R*^2^ = 0.74. Heterosexual men fixated on images of bonobos and cisgender men for a similar length of time, *p* = 0.946. They fixated on bonobos for less time than all other image categories (all *p* values < 0.001). Gay men fixated on images of bonobos for less time than all other image categories (all *p*-values < 0.001).

### Total fixation count

For heterosexual men, there was an effect of human-stimuli category on TFC, *F*(3, 184) = 88.68, *p* < 0.001, *R*^2^ = 0.59. Heterosexual men fixated on images of individuals with penises less frequently than they fixated on cisgender women, *b* = − 0.23, 95% CI (− 0.26, − 0.20), *SE* = 0.02, *p* < 0.001. They fixated on feminine trans individuals more frequently they fixated on than cisgender men, *b* = 0.11, 95% CI (0.07, 0.15), *SE* = 0.02, *p* < 0.001. Additionally, they fixated on feminine trans individuals with breasts more frequently than they fixated on feminine trans individuals without breasts, *b* = 0.13, 95% CI (0.05, 0.20), *SE* = 0.04, *p* < 0.001.

For gay men, there was an effect of human-stimuli category on TFC, *F*(3, 68) = 33.67, *p* < 0.001, *R*^2^ = 0.60. Gay men fixated on individuals with penises more frequently than they fixated on cisgender women, *b* = 0.13, 95% CI (0.08, 0.17), *SE* = 0.02, *p* < 0.001. They fixated on feminine trans individuals less frequently than they fixated on cisgender men, *b* = − 0.28, 95% CI (− 0.34, − 0.21), *SE* = 0.03, *p* < 0.001. Additionally, they fixated on feminine trans individuals with breasts and feminine trans individuals without breasts a similar number of times, *b* = − 0.04, 95% CI (− 0.16, 0.07), *SE* = 0.06, *p* = 0.439.

There was an effect of stimuli category (including control images) on TFC for heterosexual men, *F*(4, 230) = 79.87, *p* < 0.001, *R*^2^ = 0.58, and gay men, *F*(4, 85) = 45.12, *p* < 0.001, *R*^2^ = 0.68. Heterosexual men fixated on bonobos and cisgender men a similar number of times, *p* = 0.202. They fixated on bonobos less frequently than all other image categories (all *p* values < 0.001). Gay men fixated on bonobos less frequently than all image categories (all *p* values < 0.001).

### Relationship between scale measures and visual attention to human stimuli

Among heterosexual men, none of the regression models with interest in visual sexual stimuli, sociosexuality, and modern homonegativity predicting differences in responses to (1) cisgender women and individuals with penises, (2) cisgender men and feminine trans individuals, and (3) feminine trans individuals with breasts and feminine trans individuals without breasts obtained significance (*p* = 0.058–0.756; *R*^2^ = 0.03–0.16).

## Discussion

The present study examined heterosexual and gay men’s self-reported sexual attraction and visual fixations to images of cisgender men, cisgender women, feminine trans individuals with breasts, feminine trans individuals without breasts, and bonobos. In terms of heterosexual men’s self-reported sexual attraction, models’ sex and gender appeared to be relevant. Heterosexual men were most sexually attracted to cisgender women and least sexually attracted to cisgender men. They were more sexually attracted to feminine trans individuals than to cisgender men. They were also more sexually attracted to feminine trans individuals with breasts than to those without breasts. In contrast, gay men reported being most sexually attracted to cisgender men and less sexually attracted to all categories of feminine individuals.

In terms of heterosexual men’s visual attention allocation, models’ sex and gender (and species) appeared to be relevant. Namely, their attention was captured by feminine individuals quicker than by cisgender men and bonobos. Additionally, they allocated greater controlled visual attention to all feminine stimuli than to cisgender men and bonobos. However, they subsequently focused their attention on individuals who had the most female-typical qualities (i.e., cisgender women). This pattern was not exhibited by gay men.

It is worth noting that, although an effect of sex and gender were found, the effect of sex was more substantial than the effect of gender. Heterosexual men were markedly more sexually attracted to cisgender women and fixated longer on these women than on individuals with penises. Additionally, they allocated greater controlled attention to feminine trans individuals with breasts—indicating greater sexual interest in them—than to feminine trans individuals without breasts. As such, the present findings indicate that feminine trans individuals with female-typical secondary sex characteristics (i.e., breasts) draw men’s controlled attention to a greater extent than those without such characteristics.

Interest in visual sexual stimuli, sociosexuality, and modern homonegativity were not related to heterosexual men’s visual attention patterns. Additional factors that were not assessed in the present study—such as familiarity with transgender individuals over one’s life-course or societal attitudes toward transgender individuals—may influenced men’s response patterns. Subsequent research may benefit from considering whether these, or other, factors are associated with men’s visual attention patterns.

Men employ dual processes (i.e., initial automatic and controlled cognitive processes) when evaluating sexual imagery^28,29^. Physiological arousal may be elicited during the initial processing of sexual stimuli, whereas subjective arousal requires controlled attention and cognitive appraisal^30^. Cognitive appraisal incorporates explicit memory of past experience^30^. It is possible that initial processing of stimuli directs heterosexual men’s attention to stimuli that are gendered in a feminine manner and facilitates further information capture. Following this, controlled cognitive appraisal of feminine images promotes subjective sexual attraction to preferred feminine/female individuals and inhibits subjective attraction to less preferred feminine/male individuals.

These results open the possibility that heterosexual men have the capacity to experience some sexual interest in feminine trans individuals. This capacity may be enhanced or inhibited by socio-cultural factors and past experiences. In the absence of experiences that enhance subjective interest in feminine trans individuals (e.g., exposure to feminine trans individuals in sexual contexts, exposure to cultural messages that feminine trans individuals are acceptable sexual partners), this sexual interest may be inhibited. If men can access memories associating feminine trans individuals with positive sexual experiences or beliefs, then subjective sexual attraction may be elicited and fostered.

Hsu, et al.^31^ argued that attraction to feminine trans individuals with breasts is not analogous to attraction to feminine trans individuals without breasts. Feminine trans individuals without surgically augmented breasts are historical antecedents of feminine trans individuals with surgically augmented breasts^11^. The former tends to be more common in industrialized (often Western) contexts, whereas the latter tends to me more common in non-industrialized (often non-Western) contexts. Our results suggest that men perceive the two types of feminine trans individuals as distinct, but more comparable to each other, than either is to cisgender men or cisgender women. Although sexual attraction to feminine trans individuals with surgically augmented breasts may not be wholly analogous to feminine trans individuals without surgically augmented breasts, these interests are not divorced from one another and it is unlikely that they develop independently.

### Limitations and future directions

There may have been a self-selection bias in participant recruitment. It was noted in the study advertisement that participants would be required to view nude images of women, men, and transgender individuals. Men who were willing to participate may have differed from those who elected not to participate. Students who volunteer for sex research studies tend to show greater sexual openness and have more sexual experience than those who do not volunteer^32^. This self-selection bias may have been amplified because participants were required to have previously viewed nude images and be comfortable doing so.

In addition, participants were told to be mindful that they would be asked to identify and assess the sexual attractiveness of each image. They were also informed that we were interested in examining visual attention while evaluating individuals whose gender presentation varied. These instructions may have influenced participants’ attention patterns but were necessary because (1) we were interested in participants’ subjective sexual attraction to the images and (2) we needed to account for the use of an eye-tracker to the participants. To mitigate this limitation, future studies might benefit from employing a free-viewing task—in which they view images without being provided with instructions or a task to complete^33^.

Because few men report that feminine trans individuals are their preferred partners^10^, it was not possible to have the images pre-rated for sexual attractiveness. Specifically, it would not be possible to determine whether images of feminine trans individuals were rated lower than cisgender women because they were less attractive or because they were not preferred by the rater. This issue pertains to both the faces and bodies of the images.

The stimuli were not homogeneous in terms of their racial identity. It was not possible to accurately identify the racial identities of the models because the stimuli were collected online. Racial identity could have acted as a confound because some individuals show a bias in sexual/romantic interest toward members of their own race^34,35^.

This study relied on a convenience sample of Canadian undergraduate men, the majority of whom were enrolled in psychology courses. Psychology and behavior vary across human populations, and Western undergraduate students are often outliers^36^. As such, these findings are limited in their generalizability; it cannot be assumed that men in other cultures, or even other Canadian men, would respond in the same manner. A Western sample was selected because they are less likely to show appreciable sexual interest in feminine trans individuals than men in cultures in which sexual interactions between cisgender men and feminine trans individuals commonly occur^37^. Although the present study indicated that heterosexual men have the capacity to experience sexual interest in individuals who exhibit a gender presentation they prefer, but whose sex they do not, our acceptance of this finding should be tempered until it is replicated by future studies using community and non-Western samples.

The present study included a measure of homonegativity. After experiencing sexual attraction to, or engaging in sexual interactions with, feminine trans individuals, some heterosexual men question their sexual orientation^31,38^. We considered it possible that men who hold more positive attitudes toward same-sex individuals would be less concerned about experiencing sexual interests that are not stereotypically heterosexual and, in turn, would be less inhibited in their response to feminine trans individuals. However, no relationships between homonegativity and our experimental measures were found. Future studies might consider including a measure of transnegative attitudes.

Previous research has found that a portion of the men who express sexual interest in feminine trans individuals identify as bisexual or as pansexual^10^. Bisexual and pansexual men were not included in the present study because their responses to the images of cisgender women and men could not be directly compared to the responses of heterosexual men. Additional insights into the interplay between sexual orientation and sexual interest in feminine trans individuals would be garnered by comparing heterosexual, bisexual, and pansexual men’s response to feminine trans individuals.

The present findings supported the idea that models’ sex and gender were both related to heterosexual men’s visual attention allocation. The effect of sex appeared to be greater than the effect of gender. However, to further parse the effect of sex and gender on heterosexual men’s sexual responses, future studies would benefit from including stimuli of masculine transgender individuals.

## Data Availability

Data used for analysis can be accessed using the link provided in the figshare repository, https://doi.org/10.6084/m9.figshare.11620065.v1^3,39^.
